# Comparative Assessment of Neural Radiance Fields and 3D Gaussian Splatting for Point Cloud Generation from UAV Imagery

**DOI:** 10.3390/s25102995

**Published:** 2025-05-09

**Authors:** Muhammed Enes Atik

**Affiliations:** Department of Geomatics Engineering, Faculty of Civil Engineering, Istanbul Technical University, Istanbul 34469, Türkiye; atikm@itu.edu.tr

**Keywords:** photogrammetry, NeRF, gaussian splatting, point cloud, UAV, structure from motion, multiview stereo, artificial intelligence

## Abstract

Point clouds continue to be the main data source in 3D modeling studies with unmanned aerial vehicle (UAV) images. Structure-from-Motion (SfM) and MultiView Stereo (MVS) have high time costs for point cloud generation, especially in large data sets. For this reason, state-of-the-art methods such as Neural Radiance Fields (NeRF) and 3D Gaussian Splatting (3DGS) have emerged as powerful alternatives for point cloud generation. This paper explores the performance of NeRF and 3DGS methods in generating point clouds from UAV images. For this purpose, the Nerfacto, Instant-NGP, and Splatfacto methods developed in the Nerfstudio framework were used. The obtained point clouds were evaluated by taking the point cloud produced with the photogrammetric method as reference. In this study, the effects of image size and iteration number on the performance of the algorithms were investigated in two different study areas. According to the results, Splatfacto demonstrates promising capabilities in addressing challenges related to scene complexity, rendering efficiency, and accuracy in UAV imagery.

## 1. Introduction

Unmanned aerial vehicles (UAVs) are being equipped with advanced capabilities within the framework of the emerging concept of low-altitude economy [1]. UAVs integrated with different sensors are widely used in mapping, photogrammetry, and remote sensing applications due to their ability to produce three-dimensional spatial data [2,3,4,5]. In particular, camera systems integrated into UAVs provide aerial images with very high spatial resolution. Producing three-dimensional (3D) models from these images is one of the main research areas of photogrammetry. 3D reconstruction is an important technology used in computer vision and photogrammetry applications, such as quality control, reverse engineering, structural monitoring, and digital preservation [6]. Many UAV image-based 3D point cloud reconstruction methods and software have been developed in the last decade. In addition to techniques such as Structure-from-Motion (SfM) and MultiView Stereo (MVS) for UAV-based 3D modeling, remote sensing technologies such as LiDAR have recently been explored for their potential in capturing high-resolution 3D data [7].

In conventional photogrammetry-based methods, brightness information is used for feature extraction and ignored for 3D modeling. However, photogrammetry shows limitations, especially in 3D measurement of reflective and poorly textured objects [8]. For example, specular reflections in images can lead to noisy results for highly reflective and poorly textured objects, while transparent objects can present significant challenges due to texture changes caused by refraction and mirror-like reflections. With the use of artificial intelligence (AI) techniques in the production of digital 3D representations, methods such as Neural Radiance Fields (NeRF) [9] and 3D Gaussian Splatting (3DGS) [10] have been developed. NeRF and 3DGS hold great potential in 3D modeling studies since they also allow for impressively reconstructing reflectance properties beyond simple color in addition to geometry information. With NeRF, the whole scene is treated as a radiation field consisting of several beams. The volume density of every sampled point in 3D space is estimated using a neural network, and the ray walk algorithm is utilized to determine the RGB values generated by each ray in an image [11]. NeRFs utilize neural networks to generate so-called radiance fields rather than depending on the reconstruction of mathematical links between an image and the 3D world space, even though the initial point is still a collection of overlapping images [7]. However, compared with other well-established technologies such as photogrammetry, more research is needed to explore the potential of NeRFs and Gaussian Splatting, especially in UAV-based 3D modeling. Existing studies [7,12] are limited to visual interpretation and simpler point cloud comparisons. The effect of determining the appropriate parameters of NeRF and 3DGS methods on efficient and fast 3D reconstruction of architectural and natural scenes needs to be investigated comprehensively and quantitatively.

In this study, 3D point clouds of two selected study areas, which have different topographic structures in Istanbul Technical University, Ayazaga Campus, were generated with SfM-MVS as well as modern NeRF-based approach Nerfacto, 3DGS implementation Splatfacto, and Instant-NGP methods developed within the framework of Nerfstudio [13] using UAV images with very high spatial resolution. The effects of different parameter values on the performance of the algorithms were investigated. The point clouds obtained with NeRF-based and Splatfacto methods were evaluated with different analyses, such as rendering quality, multiscale model-to-model cloud comparison (M3C2) distance, and cross-section. Experiments conducted under real environmental conditions increase the practical validity of this study. Additionally, by examining different downscale factors and iteration numbers, it provides important practical implications regarding the applicability of the methods, especially in studies performed with limited hardware.

## 2. Related Works

3D reconstruction with UAV imagery is an important research area in computer vision. The photogrammetric method is still widely used in terrestrial and UAV photogrammetry. Although COLMAP [14] and MVSnet [15] methods have yielded successful results, reconstructing scenes with poorly textured areas remains a persistent challenge, and the computational time required remains high.

Unlike explicit 3D representation methods such as point clouds [16], voxels [17] or meshes [18], NeRF, which uses an implicit representation method, has brought new perspectives to image-based 3D reconstruction techniques. In the literature, methods based on NeRF are developed for various purposes. Mip-NeRF [19] and Mip-NeRF 360 [20] are two examples of NeRF techniques that have advanced significantly and solve issues like anti-aliasing and managing scenes with 360-degree camera rotation. Instant-NGP [21] is another noteworthy advancement that significantly increases rendering performance and reduces memory needs by utilizing multiresolution hash grids. Condorelli et al. [22] showed that NeRF networks can be an alternative, especially in cases where conventional photogrammetric techniques do not provide satisfactory results. Murtyioso et al. [23] compared photogrammetry and NeRF techniques using terrestrial imagery. NeRF was shown to provide 3D models with significant noise. Balloni et al. [24] conducted a comparative analysis study between NeRFs and photogrammetry to model a statue.

Recently, 3DGS has emerged as a competitive alternative to NeRF for 3D rendering reconstruction. It enables fast real-time rendering of stereo images. Several lines of research are currently interested in investigating, comparing, and integrating NeRF and 3DGS [25].

Processing of UAV imagery with NeRF and 3DGS is often used for cultural documentation [26], tree modeling [27], mapping [28], and 3D reconstruction [29,30]. Jia et al. [31] propose the Drone-NeRF framework to enhance the efficient reconstruction of unbounded large-scale scenes suited for drone oblique photography using NeRF. Li et al. [32] propose a new NeRF point sampling method created using a UAV imagery compatible with a global geographic coordinate system and suitable for a UAV view. Wang et al. [33] developed a NeRF-based orthophoto generation approach to eliminate distortions in orthophotos produced by conventional photogrammetric methods. Wei et al. [28] proposed TDOM-NeRF, a NeRF-based method. In this method, multiview UAV images are used as input, and the scene is implicitly represented using mixed grid features and multilayer perceptrons. Chen et al. [34] present a NeRF based True Digital Orthophoto Map (TDOM) generation approach called Ortho Neural Radiance Field (Ortho-NeRF). Ortho-NeRF indirectly reconstructs each tile using small tiles generated in the scene. It generates the TDOM of each tile and merges it at the end. Wu et al. [35] proposed a method based on 3DGS for 3D modeling with UAV images. The study presents a data segmentation method adapted for large-scale aerial imagery, which makes 3DGS applicable for surface reconstruction over extensive scenes. The results obtained are compared with those of traditional aerial MVS methods. Ham et al. [36] developed an approach for 3D building modeling using 3DGS results compared with those from UAV images. Qian et al. [30] proposed an improved 3DGS approach for 3D reconstruction with UAV images. Especially in large areas, it calculates the Gaussian contribution score, pruning unnecessary 3D Gaussians for the optimum solution but preserving surface details with ray tracing volume rendering. There are also methods such as Sat-NeRF [37] that have been developed to generate neural radiation fields for satellite imagery.

While 3DGS and NeRF have demonstrated potential for producing whole 3D scenes, their use is currently limited to smaller objects and scenarios. More study is required to expand their capacities for more complex and diverse environments.

## 3. Materials and Methods

### 3.1. Study Area and Data Capturing

**Figure 1:**
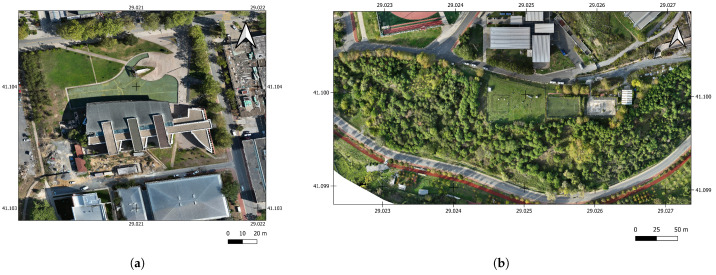
Orthophoto of the study areas: (**a**) Study area 1. (**b**) Study area 2.

In this study, two study areas with different topographic features were selected in the Istanbul Technical University Ayazağa Campus. The first study area is the Mustafa İnan Library and its environment (Figure 1). The study area covers an area of approximately 140 m × 140 m containing impervious surface, vegetation, and water. The second study area is in an area that includes mostly dense forest. During the flight with the UAV, 560 oblique and nadir angle images for the first study area and 272 nadir images for the second study area were obtained in RTK mode.

The DJI Mavic 3M RTK UAV (Dà-Jiāng Innovations Science and Technology Co. Ltd., Shenzhen, China) was used to capture aerial images. The DJI Mavic 3M’s lens features FOV: 84°, an equivalent focal length of 24 mm, aperture of f/2.8 to f/11, and a focus of 1 m to *∞*. The flight altitude is 35 m relative to the take-off point. The flight parameters were selected with an 80% overlap ratio and a 70% sidelap ratio. The ground sampling distance (GSD) is 0.8 cm/pixel.

### 3.2. Neural Radiance Fields (NeRF)

NeRF was first proposed by Mildenhall et al. [9]. They created a deep learning model and a ray-tracing algorithm for the brightness field representation of the scene. This method is capable of generating 3D views of complex scenes by optimizing a continuous scene function from oriented images. The volume is supposed to serve as a differential opacity that shows how much radiance a ray gathers as it passes through each point. The inputs of NeRF are a set of continuous 5D coordinates consisting of spatial locations **x** = (x, y, z) and viewing directions **d** = (θ, δ) and whose outputs are the volume density (σ) in each direction and the emitted brightness **c** = (r, g, b) depending on the viewing direction. NeRF presents a static 3D scene as a continuous 5D function:

(1) $$F_{\Theta }:\left(x,d\right)\to \left(c,\sigma \right)$$

$F_{\Theta }$ is a multilayer perceptron (MLP) which is a feed-forward neural network that outputs color information **c** and volume density σ. The viewing direction does not affect σ, but c is determined depending on both the viewing direction and coordinates. $F_{\Theta }$ has 8 fully connected layers and processes the 3D coordinate **x**. The output of $F_{\Theta }$ is a feature vector of σ and 256 dimensions. The camera ray’s viewing direction is added to this feature vector and passed to a fully connected layer that outputs the view-dependent RGB color. The differential probability of a ray terminating at an infinitesimal particle at location x can be described as volume density σ(x). The expected color of the camera ray r(t)=o+td with near and far bounds $t_{n}$ and $t_{f}$ is calculated by Equation (2).

(2) $$C\left(r\right)=\int_{t_{n}}^{t_{f}}T\left(t\right)\sigma \left(r\left(t\right)\right)c\left(r\left(t\right),d\right)dt,\;\mathrm{where}\;T\left(t\right)=\exp\left(-\int_{t_{n}}^{t}\sigma \left(r\left(s\right)\right)ds\right)$$

The function T(t) is defined as the probability that the beam travels from $t_{n}$ to *t* without hitting another particle. A view is rendered in the continuous NeRF by estimating the integral C(r) for a camera ray passing through each pixel of the virtual camera.

When the ray is divided into N equally spaced bins and a sample is drawn uniformly from each bin according to the non-deterministic stratified sampling approach, the equation is transformed into Equation (3).

(3) $${\hat{C}}_{\left(r\right)}\approx \sum_{i=1}^{N}\alpha_{i}T_{i}c_{i}$$

In this study, Instant-NGP and Nerfacto [13] models developed within the Nerfstudio framework were used. Instant-NGP [21] uses multilevel hash coding and specific spatial hash functions, significantly improving training speed and processing quality with fewer parameters than NeRF. Compared with previous NeRF acceleration techniques such as octree, the hash table requires fewer if branches to store weights, which helps improve GPU parallel efficiency. The objective is to associate coordinates with trainable feature vectors that may be refined within the conventional framework of NeRF training. The fundamental concept of the enhanced sampling approach is to exclude sampling in vacant spaces and to disregard sampling in regions of high density. This is achieved by maintaining a set of multiscale occupancy grids that roughly demarcate empty and non-empty space. Occupancy is represented by a single bit, and a sample on a ray is omitted if its occupancy is insufficient. The occupancy grids are kept separately from the trainable encoding and are revised during training according to new density estimations. Furthermore, the hash table is particularly favorable to memory access, with an O(1) query operation time complexity.

Pose refinement is the first crucial domain where Nerfacto outperforms the base approach. When image poses are incorrect, the rebuilt scene loses sharpness and has foggy artifacts. Nerfacto technique optimizes the postures for every training iteration using the back-propagated loss gradients. In ray sampling, light rays are modeled as conical frustums. At a certain distance from the camera origin, the rays are equally sampled in the piecewise sampling step. After that, consecutive parts of the conical ray are sampled at progressively larger step sizes. This ensures that close parts of the scene are sampled in high detail while distant objects are sampled efficiently. The output is sent into a bid sampler that merges the selected positions into the parts of the scene that most significantly impact the final 3D scene rendering. The production of sample steps is input into the Nerfacto space, which includes view embedding to account for differing exposure between training images [13].

### 3.3. 3D Gaussian Splatting (3DGS)

Kerbl et al. [10] presented 3DGS, which compresses 3D scene information and uses a 3D anisotropic Gaussian kernel. Each 3D anisotropic Gaussian kernel contains a learnable mean, opacity, and covariance matrix.

Gaussian splatting’s efficient rasterization and exceptional 3D Gaussian image processing quality make it easy to integrate with virtual reality (VR) technologies. By utilizing 3DGS technology, more realistic and intricate image rendering effects can be obtained in VR environments.

**Figure 2:**
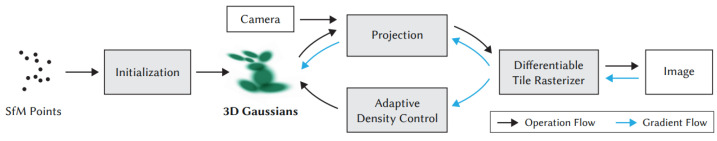
The rendering flow of 3DGS [10].

Three-dimensional Gaussian Splatting has four main steps (Figure 2): (1) Obtain an initialized sparse point cloud generated with SfM-MVS. (2) Create 3D Gaussian ellipsoid sets utilizing a sparse point cloud. (3) Render a new 2D view. (4) Optimize scene representation.

In the first step, SfM is employed to estimate the interior and exterior camera parameters, as well as relative positions and orientations of the images, based on epipolar geometry. Open-source COLMAP [14] is generally used for this purpose. This method avoids unnecessary calculations in free space while preserving the advantageous characteristics of the continuous volume radiation field for scenario optimization. It provides the fundamental information needed for subsequent scene reconstruction, thus improving the precision and reliability of the structural recovery process. Secondly, 3DGS calculates the mean values of a set of 3D Gaussian random variables, which are the center locations of the Gaussian ellipsoid, using the sparse point cloud. Each Gaussian random variable can be described in terms of a covariance matrix with mean (μ) and probability (Σ) density (Equation (4)).

(4) $$f\left(x|\mu ,\Sigma \right)=\frac{1}{\sqrt{{\left(2\pi \right)}^{3}\left|\Sigma \right|}}\exp\left(-\frac{1}{2}{\left(x-\mu \right)}^{T}\Sigma^{-1}\left(x-\mu \right)\right)$$

where the covariance matrix Σ refers to the rotation matrix R of the Gaussian ellipsoid and the scaling matrix S in each axis.

In 3DGS, the spherical harmonic function is used to color the point cloud, which makes the point cloud display different colors at different angles.

### 3.4. Structure from Motion (SfM)

The SfM technique allows an object or structure to be reconstructed in three dimensions from the movement of a camera. Here, ‘motion’ refers to camera movement/displacement, and ‘structure’ refers to both the object and its geometry. Generally accepted approaches use a combination of image processing algorithms, robust orientation methods, bundle adjustment with self-calibration, stereo image analysis, and point cloud processing [38].

The SfM does not need any prior knowledge before the scene is reconstructed. The camera pose and scene geometry are simultaneously reconstructed by automatically detecting corresponding features in stereo images [2]. Using a feature detector like SIFT [39], the algorithm’s initial step is to find matching points across several stereo pictures. In SfM, feature-based techniques are frequently employed for the image matching process. These techniques are robust to changes in illumination, perspective, and homography [40]. The fact that these features are invariant under radiometric and geometric changes allows SfM to identify them uniquely across multiple images. The intrinsic projective geometry between two views, known as epipolar geometry, is used to evaluate matching features between the two images. In Equation (5), the epipolar geometry is expressed using a 3 × 3 matrix, which is known as the fundamental matrix (*F*).

(5) $$x^{\prime }Fx=0$$

where $x^{\prime }$ and *x* are the corresponding points on the stereo images. They are projected points of a 3D point onto the camera image plane [41]. The normalized eight-point approach is used to match features in order to compute the fundamental matrix in a preliminary approach.

Finding scene overlap in the input images (*I*) is the first step of the correspondence search procedure. For each image ($I_{i}$), SfM detects feature sets $F_{i}=\left(x_{j},f_{j}\right)|j=1...N_{F_{i}}$ containing the features specified by an appearance descriptor ($f_{j}$). SfM aims to identify images belonging to the same scene using extracted features. The naive approach uses a similarity metric to detect identical features and compute the overlap of images. For each feature in image $I_{b}$, it searches for feature correspondences by finding the most similar feature in image $I_{a}$. The matched images need to be geometrically verified. If a valid transformation maps a sufficient number of features between images, they are considered geometrically verified. For this purpose, robust estimation techniques such as RANSAC are usually preferred. The camera’s position and orientation are estimated for each image by solving the perspective-n-points (PnP) problem. Finding a calibrated camera’s position given a collection of 3D points in the environment and the 2D projections of those points in the image is known as the PnP problem [14].

Bundle adjustment is a non-linear joint refinement of the camera parameters $P_{c}$ and the point parameters $X_{k}$ that aims to minimize the distance between the projected and observed points iteratively. This method is used on the collective set of camera and scene parameters:

(6) $$E=\sum_{j}\rho_{j}\left({∥\pi \left(P_{c},X_{k}\right)-x_{j}∥}_{2}^{2}\right)$$

Using a function π that projects scene points to image space. and a loss function $p_{j}$ to potentially downweight outliers. $x_{j}$ is the observed image point. Several images are used in this iterative approach to gradually improve camera position and orientation parameters [14].

### 3.5. Evaluation Metrics

The quality of the rendering was evaluated with Peak Signal-to-Noise Ratio (PSNR) [42], Structural Similarity Index Measure (SSIM) [43], and Learned Perceptual Image Patch Similarity (LPIPS) [44]. Lossy information related to the generated image is indicated by PSNR, as shown in Equation (7). Here, MAX refers to the maximum possible pixel value, the Mean Squared Error (MSE) is the difference between each pixel, averaged across all pixels.

(7) $$\mathrm{PSNR}=10\log\frac{MAX^{2}}{MSE}$$

SSIM is a metric that evaluates human-perceived image quality rather than a quantitative error. It is calculated by evaluating the brightness, contrast, and structure between the images are considered for calculating SSIM. In Equation (8), $\mu_{x}$ and $\mu_{y}$ refer to the average pixel values of the images, $\sigma_{x}$ and $\sigma_{y}$ represent the standard deviations of the pixel values, and $\sigma_{xy}$ is the covariance between the pixel values of the images.

(8) $$\mathrm{SSIM}\left(x,y\right)=\frac{\left(2\mu_{x}\mu_{y}+C_{1}\right)\left(2\sigma_{xy}+C_{2}\right)}{\left(\mu_{x}^{2}+\mu_{y}^{2}+C_{1}\right)\left(\sigma_{x}^{2}+\sigma_{y}^{2}+C_{2}\right)}$$

LPIPS is a model that assesses image similarity by simulating human perceptual characteristics using AlexNet, VGG, and SqueezeNet. It measures the similarity between two features by leveraging the middleware features of networks trained on the ImageNet data set. In Equation (9), where *x* and $x_{0}$ are input features, *l* represents the layer, *y* and $y_{0}$ are unit-normalized feature vectors at the channel level, ω is a scaling factor, and *H* and *W* are the height and width, respectively [45].

(9) $$\mathrm{LPIPS}\left(x,x_{0}\right)=\sum_{l}\frac{1}{H_{l}W_{l}}{∥\omega_{l}⊙\left(\tilde{y_{h\omega }^{\prime }}-\hat{y_{0h\omega }^{\prime }}\right)∥}_{2}^{2}$$

## 4. Results

This study investigates the 3D modeling performance of the Nerfacto, Instant-NGP, and Splatfacto methods developed using the Nerfstudio framework with UAV images. The SfM method was implemented via the DJI Terra (version 4.4.0) software. For Nerfacto, Instant-NGP, and Splatfacto, camera coordinates must be calculated with SfM. For pose estimation and sparse point cloud generation, the SfM module in COLMAP was used. With this approach based on epipolar geometry, camera pose estimation of UAV images was realized. These values were used as input for NeRF-based methods (Nerfacto and Instant-NGP) and Splatfacto. Accuracy evaluation was performed concerning the point cloud produced with SfM-MVS. Point clouds generated with all methods were filtered, and noisy points were eliminated. Filtering was applied using the Statistical Outlier Removal (SOR) tool in CloudCompare (version 2.13.2) software. SOR is a filtering method based on a statistical method. SOR assumes that the points in the point cloud have a Gaussian distribution and evaluates whether the deviation from the distance points is an outlier according to the standard deviation. If the distance of a point deviates from the average distance by more than the threshold value, it is considered an outlier and removed [46]. The determining parameter in SOR is the number of neighborhood points. This value is generally expected to be between 1 and 6. Ineffective filtering and high computational load may occur when the parameter values exceed 6. In this study, the optimum number of neighbor points for both study areas was determined as 6. Experiments were carried out on NVIDIA GeForce GTX 4080 GPU (NVIDIA, Santa Clara, CA, USA) and 64 GB RAM.

### 4.1. Accuracy Assessment of Photogrammetric Point Clouds

**Figure 3:**
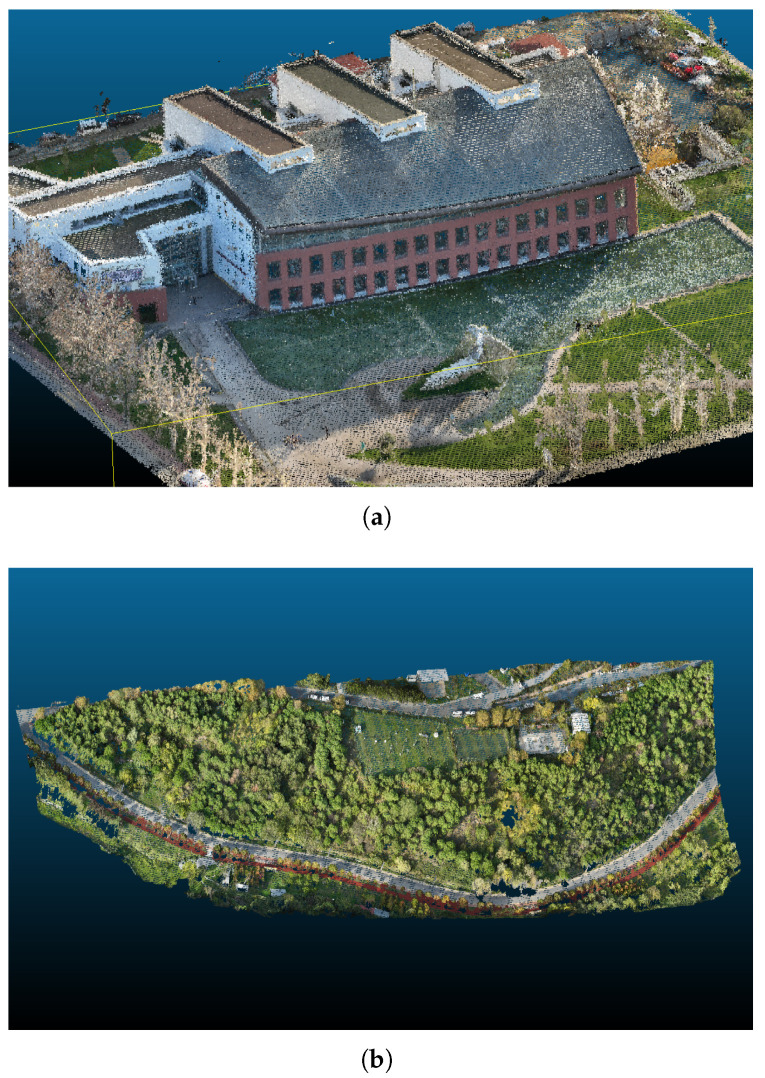
Point clouds generated with SfM-MVS and used as reference: (**a**) Study area 1 (number of points: 10,003,202). (**b**) Study area 2 (number of points: 24,098,641).

Since the SfM-MVS model is the reference for comparison, accuracy assessment was performed using GCP. Camera positions are defined in the TUREF TM30 coordinate system derived from RTK data. To evaluate the positioning performance of the RTK module integrated into the UAV and to analyze the accuracy of SfM-MVS point clouds, the coordinates of 9 GCPs in study area 1 and 8 GCPs in study area 2 were obtained by terrestrial surveying with a GNSS receiver. SfM-MVS operation and accuracy evaluation were performed with DJI Terra software. GCP error assessments are presented in Table 1 and Table 2. Point clouds generated with SfM-MVS are presented in Figure 3.

### 4.2. Assessment of Rendering Quality

The effect of the number of iterations in Nerfacto, Instant-NGP, and Splatfacto methods was investigated. Experiments were repeated with 5K, 30K, and 50K iterations. These values were determined considering previous studies [12]. Additionally, since UAV images have large sizes, they are difficult to process with existing hardware. For this reason, downscale is applied to the images at specific rates. The effect of image reduction was also investigated in this study. The images were reduced by 4 and 8. While Nerfstudio offers the option to adjust the number of points in the derived point cloud for Nerfacto and Instant-NGP, 3D Gaussian Splatting does not offer this adjustment and produces an automatically generated point cloud.

**Figure 4:**
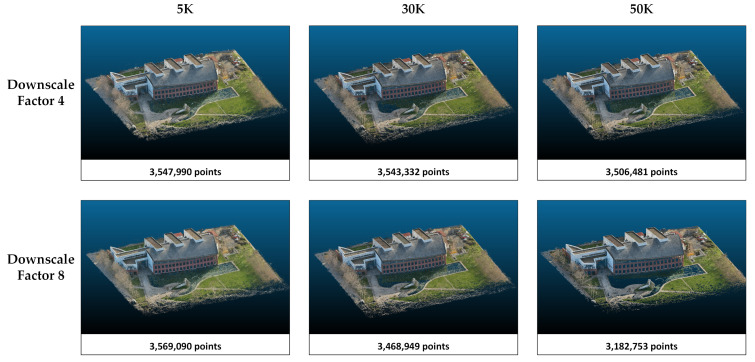
The point clouds generated with Nerfacto for study area 1. Point clouds are shown separately according to the image size and iteration number parameters.

**Figure 5:**
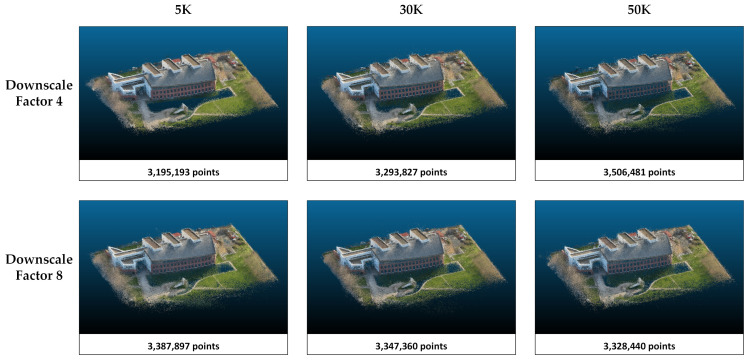
The point clouds generated with Instant-NGP for study area 1. Point clouds are shown separately according to the image size and iteration number parameters.

**Figure 6:**
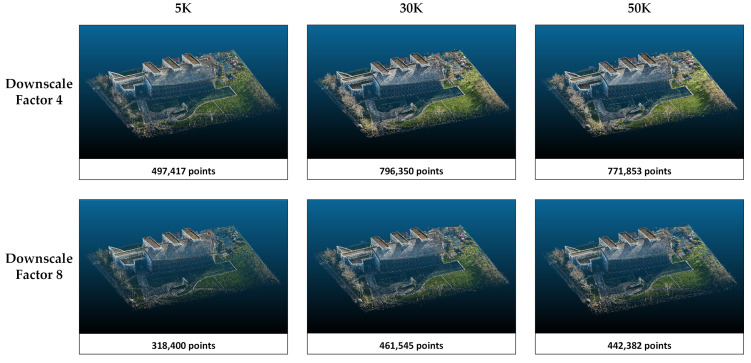
The point clouds generated with Splatfacto. Point clouds are shown separately according to the image size and iteration number parameters.

The rendering performances of the methods in study area 1 are compared according to the PSNR, SSIM, and LPIPS metrics (Table 3). Higher values in PSNR and SSIM indicate better image reconstruction quality, while a lower LPIPS suggests a closer perceptual resemblance to the ground truth. According to the results presented in Table 1, PSNR and SSIM increase, and LPIPS decreases with increasing downscale in Splatfacto and both NeRF algorithms. In other words, the quality of the model reconstruction and the perception of realism increase when the image size is reduced. However, Nerfacto and Instant-NGP achieve the highest PSNR and SSIM values with 30K iterations. At 50K iterations, there is a slight decrease in these metrics. The 5K iterations are insufficient compared with 30K and 50K. When Splatfacto is applied, metric values improve as the number of iterations increases. Especially when comparing 5K and 50K iterations, PSNR increases from 20 to 23. Similarly, the SSIM value increases by about 0.17. Among all three methods, Splatfacto provided the most successful metrics. Although Nerfacto has the lowest metrics, its LPIPS values are close to Instant-NGP. Point clouds generated by the three methods are presented in Figure 4, Figure 5 and Figure 6.

**Figure 7:**
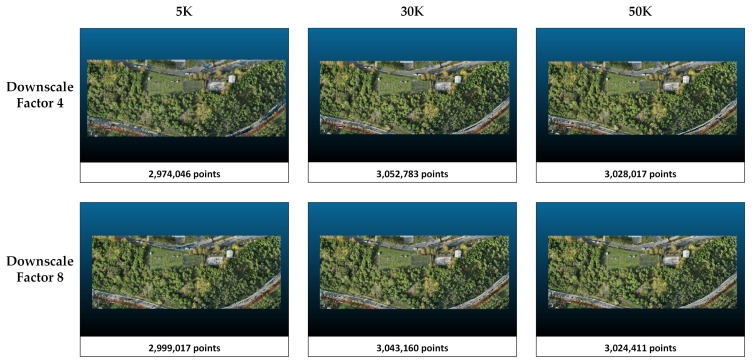
The point clouds generated with Nerfacto for study area 2. Point clouds are shown separately according to the parameters of image size and iteration number.

**Figure 8:**
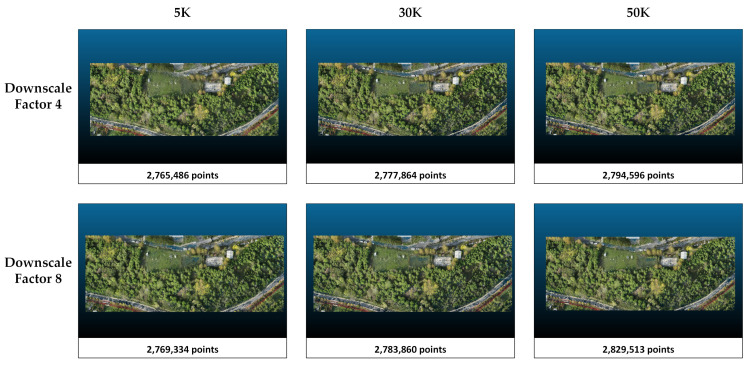
The point clouds generated with Instant-NGP for study area 2. Point clouds are shown separately according to the parameters of image size and iteration number.

**Figure 9:**
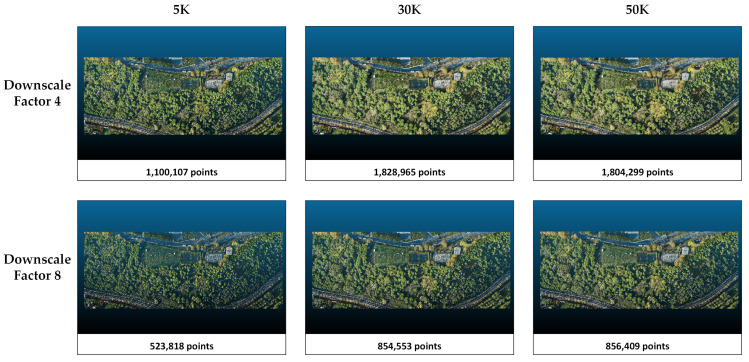
The point clouds generated with Splatfacto for study area 2. Point clouds are shown separately according to the image size and iteration number parameters.

The metrics obtained in study area 2 are presented in Table 4. When the downscale factor is applied as 8, there is an improvement in the metrics. In Splatfacto, when the downscale is set to 8, the number of points in the point cloud is reduced by about 50% compared with when the downscale factor is set to 4. With respect to the number of iterations, more training in Nerfacto and Instant-NGP only marginally increases the number of points and only slightly improves the metrics. On the other hand, increasing the number of iterations from 5K to 30K increases the number of points by more than 60%. However, there is no significant difference between the point clouds obtained when 30K and 50K iterations are applied. According to Table 4, Splatfacto performs better than Instant-NGP and Nerfacto in study area 1. The most successful metrics (PSNR = 28.9315, SSIM = 0.9180, and LPIPS = 0.0561) are obtained with Splatfacto when downscaled to 8, and the 50K iteration configuration is chosen. The lowest metrics are obtained in the Nerfacto method with a downscale factor of 4 and 5K iterations. PSNR, SSIM, and LPIPS values are 19.9150, 0.4104, and 0.6216, respectively. The overall successful method is clearly Splatfacto, while Nerfacto has the lowest metrics in comparison. The point clouds and number of points produced by each method in study area 2 are presented in Figure 7, Figure 8 and Figure 9.

### 4.3. M3C2 Distance Analysis

The point clouds generated from Nerfacto, Instant-NGP, and Splatfacto were compared with those generated in DJI Terra (version 4.4.0). For this purpose, the six models with the highest metric values in each study area were analyzed. The results of the comparison were performed using open-source CloudCompare (version 2.13.2) software.

Point cloud registration was performed using the point cloud generated by SfM-MVS as a reference, ensuring all point clouds aligned within a common coordinate system. A two-step registration process is performed to align two point clouds. The first step is coarse registration, and the second step is fine registration. In coarse registration, a 3D conformal transformation is applied using conjugate points. While at least three conjugate points are used for the transformation, five conjugate points are used in this study. As a result of this transformation, rotation, translation, and uniform scale values are calculated between the point clouds. The coarse registration is provided from the aligned point cloud to the reference point cloud. The iterative closest point (ICP) method was applied for fine registration. ICP iteratively refines the alignment by minimizing the distance between the closest points in the two clouds. Although it requires intensive calculation, it is widely preferred because it is easy to apply and gives high-accuracy results [47]. The method is based on finding the transformation parameters with the closest point pairs by considering the reference point set and the survey point set to be of the same scale. The transformation parameters between the two point clouds are calculated iteratively through the relations between the conjugate points until a sufficient prescription is achieved [48]. Choosing appropriate ICP parameters plays a significant role in the method’s success. Inappropriate settings can degrade the initial alignment achieved through the conformal transformation. Multiscale model-to-model cloud comparison (M3C2) [49] calculation was performed between the transformed point clouds. M3C2 measures the distance along a local normal vector, which it calculates using the neighbourhood of each point, thus considering the local surface orientation in the distance calculations. M3C2 does not require any preprocessing steps that affect the structure of the point cloud and works directly on point clouds. Thus, the correspondence of the point clouds produced with point NeRF-based methods and Splatfacto with the photogrammetric point cloud was analyzed. NeRF and 3D Gaussian Splatting produce point clouds in a local coordinate system derived from COLMAP. After the registration stages, these point clouds were converted to the TUREF-TM30 reference coordinate system (the coordinate system of the SfM-MVS point cloud). No upsampling was applied before the registration process. All point clouds were evaluated under the same experimental conditions. The mean error and standard deviation (σ) values from an M3C2 analysis were used to assess geometric accuracy.

**Figure 10:**
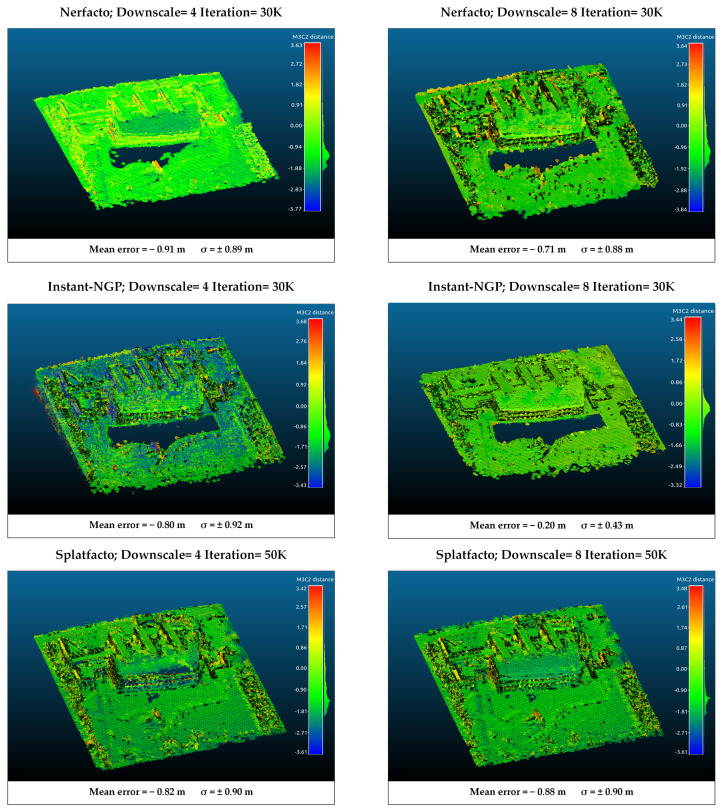
M3C2 analysis of point clouds for study area 1 using SfM-MVS point cloud as reference. Red represents positive differences, while blue represents negative differences. The unit in the legend is m.

According to the results in study area 1, the positive difference is high on the vertical surfaces of the point clouds produced with Nerfacto. On the other hand, there are generally negative errors. When the downscale factor 4 or 8 is selected, the average distance difference is around 1.3 m. When downscale is applied to point clouds produced with Instant-NGP as 4, the distance difference with the reference point cloud increases. However, when downscale is determined as 8, high compatibility with the reference surface is achieved. On the other hand, as seen in Figure 9, the information loss after filtering is higher in the Instant-NGP model with a downscale of factor 8. These losses are clearly seen in the border regions. The point clouds produced with Splatfacto have deviations similar to those of other methods. Although an average error of 1.35 m is obtained, a more holistic model is presented. Deviation generally increases toward the border regions. It is especially evident in irregular structures such as trees. Models produced with Splatfacto on flat surfaces have lower deviations. A comparison of each model with SfM in study area 1 is presented in Figure 10.

**Figure 11:**
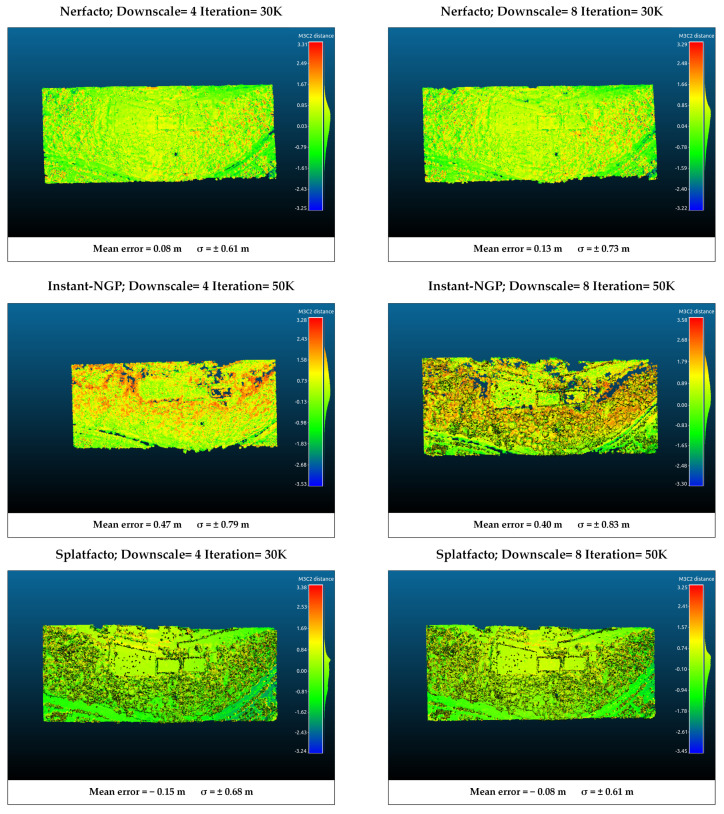
M3C2 analysis of point clouds for study area-2 using SfM-MVS point cloud as reference. Red represents positive differences, while blue represents negative differences. The unit in the legend is m.

According to the analysis results performed in study area 2, Nerfacto has an average error of approximately 0.35 m in the model selected as downscale 4, while the error increases when downscale 8 is selected. In both models produced with Instant-NGP, a significant difference in the positive error is seen. Since Instant-NGP generally produces noisier point clouds, a positive error in the distance analysis is an expected result. These differences occur especially in the wooded area. Splatfacto provides more compatible results with the reference point cloud. Although there are positive differences when downscale 8 is selected, the average error amount is around 0.2 m. The comparison results in study area 2 are presented in Figure 11.

### 4.4. Cross-Section Analysis

**Figure 12:**
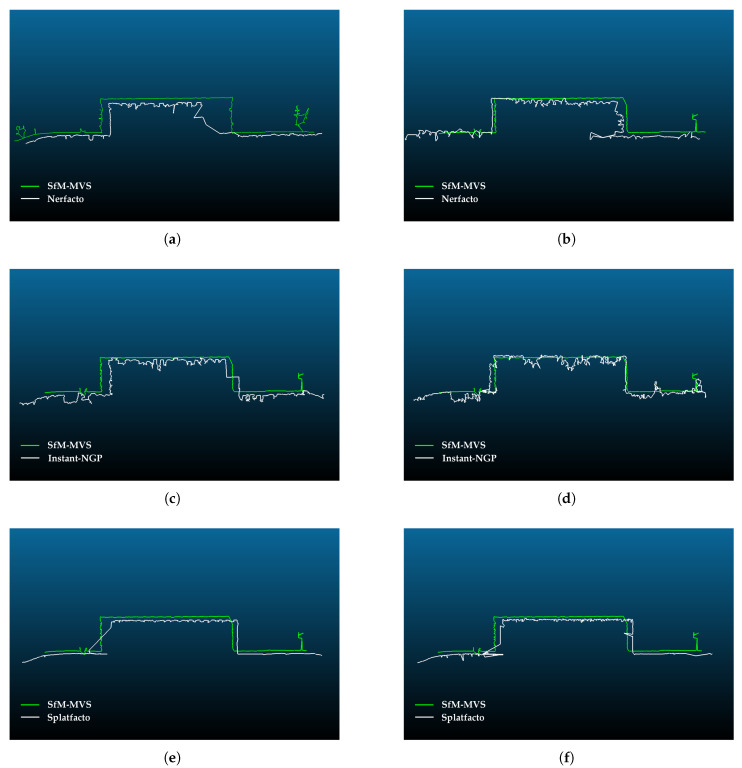
Comparison of point clouds generated by Nerf-based methods and Splatfacto with point clouds generated by SfM-MVS along a cross-section in study area 1: (**a**) Comparison of Nerfacto (downscale factor: 4, iteration: 30K) and SfM. (**b**) Comparison of Nerfacto (downscale factor: 8, iteration: 30K) and SfM. (**c**) Comparison of Instant-NGP (downscale factor: 4, iteration: 30K) and SfM. (**d**) Comparison of Instant-NGP (downscale factor: 8, iteration: 30K) and SfM. (**e**) Comparison of Splatfacto (downscale factor: 4, iteration: 50K) and SfM. (**f**) Comparison of Splatfacto (downscale factor: 8, iteration: 50K) and SfM.

Furthermore, the models were compared by extracting profiles from the point cloud generated with Nerfacto, Instant-NGP, Splatfacto, and SfM. In study area 2, since the model produced with SfM is less noisy, the profile consists of straight lines. In other models, lines are distorted due to noise. When downscale is selected as 8, the resulting shape is seen to be more compatible with SfM. In addition, Splatfacto offers the most similar and closest model to SfM. Models produced with Nerfacto contain more inconsistency than those produced with Instant-NGP and Splatfacto. The inconsistency in the Nerfacto model is especially noticeable on the left edge of the library. Cross-section comparison is presented in Figure 12.

**Figure 13:**
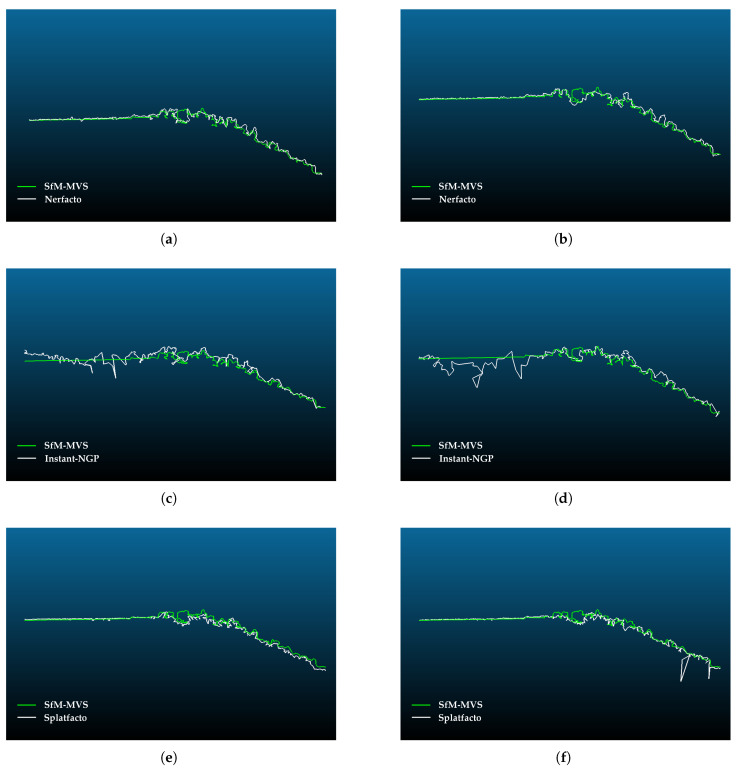
Comparison of point clouds generated by Nerf-based methods and Splatfacto with point clouds generated by SfM-MVS along a cross-section in study area 2.: (**a**) Comparison of Nerfacto (downscale factor: 4, iteration: 30K) and SfM. (**b**) Comparison of Nerfacto (downscale factor: 8, iteration: 30K) and SfM. (**d**) Comparison of Instant-NGP (downscale factor: 4, iteration: 30K) and SfM. (**d**) Comparison of Instant-NGP (downscale factor: 8, iteration: 30K) and SfM. (**e**) Comparison of Splatfacto (downscale factor: 4, iteration: 50K) and SfM. (**f**) Comparison of Splatfacto (downscale factor: 8, iteration: 50K) and SfM.

In the section taken in study area 2, the agreement with the reference point cloud (SfM-MVS) is generally high (Figure 13). The results of the noise generated in the Instant-NGP modeling of the wooded areas are also seen in the cross-section. The incompatibility increases especially in the flat areas of the land. The difference is low in the cross-sections taken from the point clouds produced with Nerfacto and Splatfacto. It can be seen that some trees cannot be modeled sufficiently in the selected section. When the downscale factor is applied as 8 in all methods, the incompatibility in the cross-sections increases.

## 5. Discussion

Considering the results of this research, comparing NeRFs, Splatfacto, and photogrammetry can be framed around several essential factors, including modeling performances, point cloud analyses, and possible conversion to other forms of representation. The common feature between these techniques is the reconstruction of interior and exterior camera orientation. NeRF-based and Splatfacto algorithms have proven to be effective for constructing 3D models from aerial images. A comparison of rendering performances reveals that Splatfacto offers superior success in 3D modeling compared with Nerfacto and Instant-NGP (Table 3 and Table 4). Splatfacto requires less computational load thanks to the splats it calculates at each point and provides successful results in complex scenes and large data sets. Generally, when the downscale factor increases, the rendering quality metric values become better. When image resolution is reduced, image detail is reduced, and errors become less noticeable. In other words, downsampling reduces the salience of fine details. In this case, the differences between the image generated by NeRF and the real image are less visible. This leads to an improvement in metric values. However, when the downscale factor is set to 8, the noise in the point clouds increases, especially in irregular structures such as trees, noisy structures become apparent. This reduces the model’s ability to represent reality. For this reason, it may not be sufficient to evaluate only the rendering metrics. Photogrammetry is an effective 3D modeling method for solid and opaque surfaces, but it has limitations due to optical effects on transparent surfaces such as glass and water. Problems such as reflection, refraction, and texturelessness prevent these surfaces from being modeled accurately. Therefore, NeRF and Splatfacto offer a superior solution compared with photogrammetry for modeling transparent surfaces. Dynamic surfaces, especially water, reduce the consistency of photogrammetric modeling due to constantly changing reflection and refraction patterns. Transparent surfaces, such as glass and water, often do not have a distinct texture, making it difficult for algorithms to detect adequate matching points. Reflections on glass surfaces can create inaccurate surface geometries in photogrammetric models.

While terrestrial image applications are typical in the literature, studies using UAV images are limited. It is challenging to capture volumetric representation in flight planning, consisting of strips for photogrammetric modeling. On the other hand, because UAV images are large-sized files, processing the images requires high hardware features. In the Nerfstudio framework, the whole image size could not be processed with the current hardware. Applying downscaling solves this problem. Similar to previous studies [7], the results show that successful 3D models can be produced when the appropriate downscale factor is applied.

According to the M3C2 analysis for the first region (Figure 10), it is seen that the lowest average error is obtained with Instant-NGP with downscale factor 8. On the other hand, when the noise status of the produced model is taken into account, the reason for this situation is that a large number of points are eliminated during filtering, and backward-compatible points remain. However, the disadvantage of this is that information loss occurs, especially in border regions and wooded areas, as seen in Figure 10. In the models produced with Splatfacto, it is seen that surfaces such as water can be modeled, although there is a higher error. In study area 2 (Figure 11), the compatibility of Slatfacto with the reference point cloud is more clearly seen. It is seen that the point clouds produced from nadir images have higher accuracy. The noisy structure of Instant-NGP caused positive errors in the M3C2 analysis. The noise in the point cloud also affects the distance calculation. In this context, we can evaluate the Nerfacto (Downscale: 8) model as an error caused by the noise. Additionally, since the coordinate transformation is applied to the models by taking the model produced with SfM as a reference, the GCP selection also has an effect. However, since the same five GCPs are used for the transformation of all models, the selection effect of the GCP is minimized. If there is no reference 3D model (image coordinates cannot be obtained precisely with RTK), GCP coordinates are determined by terrestrial measurements (e.g., GNSS receiver), and georeferencing is performed. Thus, all point clouds generated with SfM-MVS, NeRF-based methods, and 3DGS are obtained in the specified reference coordinate system.

According to Figure 12, Slatfacto has a more accurate result in terms of geometry. Instant-NGP uses fewer samples to produce faster results than the basic NeRF algorithm, which increases the noise. It is observed that the point cloud becomes noisier in Nerf-based methods as the image resolution decreases, and this is in accordance with the findings in the literature [7]. Splatfacto gives a more compatible result with the reference point cloud along the cross-section. For Instant-NGP, a distorted section line occurs due to noise. Since there are more trees in study area 2, the fit is a bit more challenging to determine (Figure 13). However, similar to study area 1, it is seen that Instant-NGP provides a more mismatched line. Nerfacto and Splatfacto have almost identical results. It is seen that some trees are missing in the point cloud model.

When considering the time required for processing data, photogrammetry, as expected, requires more processing time for point cloud generation. Although Nerf-based methods are relatively fast and can build most of the model in a few minutes, they are slow compared with Splatfacto. In both stıdy areas, Nerf-based methods complete 50K iterations in 15 min, while Splatfacto completes in 4 min. In the test, there is a significant time difference between Nerf-based methods and Splatfacto. Nerf-based methods complete the test in 22 s, while Splatfacto completes the test in 1 s. The test time is independent of the number of iterations. However, the processing time of Nerf and Splatfacto is also highly dependent on the size of the input image. For all algorithms, when the downscale factor 4 is selected, training and testing times are doubled compared with when the downscale factor 8 is selected.

## 6. Conclusions

In this study, the performances of photogrammetry and NeRF-based and 3DGS methods in 3D point cloud production with UAV images were compared. The Nerfacto, Instant-NGP, and Splatfacto methods developed within the Nerfstudio framework were used. According to the results, it is understood that NeRF and 3DGS have promising results as an alternative to photogrammetry when appropriate parameters (number of iterations and input image size) are selected. As a result of the study, although photogrammetry still maintains its power in 3D model production in UAV images, the use of 3DGS is recommended for UAV-derived point cloud generation. Considering the processing times, Splatfacto and NeRF methods are becoming important for UAV image processing, especially in emergency situations where the rapid acquisition of comprehensive measurement data is critical.

Although 3DGS and NeRF-based methods provide realistic models, technical constraints such as memory consumption and resolution optimization were identified as challenges to be overcome during the study process.

The results show that the methods offer different advantages according to their application areas and reveal the potential benefits of hybrid approaches. In the future, the combined use of NeRF and photogrammetry methods will complement the limitations of both methods. They can be used together in many areas, such as the more comprehensive and accurate representation of cultural heritage objects. Additionally, it is planned to integrate GNSS data into the method to solve the georeferencing problem in the NeRF and 3DGS methods.

## Figures and Tables

**Table 1:** Accuracy assessment of SfM-MVS process for study area 1. All GCPs are defined as check points.

Point ID	Error X (m)	Error Y (m)	Error Z (m)
PN1	−0.028	0.002	−0.141
PN2	−0.026	0.067	−0.154
PN3	−0.01	0.014	−0.066
PN4	−0.02	0.036	−0.065
PN5	−0.046	0.057	−0.112
PN6	0.002	0.025	−0.111
PN7	−0.03	0.033	−0.13
PN8	−0.034	−0.017	−0.116
PN9	−0.010	0.003	−0.268
**RMSE**	**0.026**	**0.035**	**0.141**

**Table 2:** Accuracy assessment of SfM-MVS process for study area 2. All GCPs are defined as check points.

Point ID	Error X (m)	Error Y (m)	Error Z (m)
PN1	−0.019	0.018	0.068
PN2	−0.044	0.015	0.187
PN3	−0.015	0.002	0.209
PN4	0.009	−0.029	0.175
PN5	−0.016	0.01	0.187
PN6	−0.007	0.023	0.16
PN7	−0.004	−0.017	0.372
PN8	0.015	−0.009	0.359
**RMSE**	**0.020**	**0.017**	**0.240**

**Table 3:** Comparison of the algorithms based on the rendering quality metrics in study area 1.

Method	Number of Iterations	Downscale (4)			Downscale (8)		
		**PSNR**	**SSIM**	**LPIPS**	**PSNR**	**SSIM**	**LPIPS**
	5K	16.1579	0.4472	0.7103	16.9832	0.4814	0.4958
Nerfacto	30K	16.3759	0.4533	0.5684	16.9837	0.4826	0.3699
	50K	16.2834	0.4431	0.5416	16.8934	0.4773	0.3426
	5K	18.5218	0.5394	0.6403	18.8985	0.6161	0.4424
Instant-NGP	30K	18.9743	0.5937	0.5194	19.6127	0.6848	0.3363
	50K	18.7872	0.5961	0.5078	19.3977	0.6878	0.3236
	5K	20.5129	0.6644	0.4734	21.1858	0.7182	0.3325
Gaussian Splatting	30K	23.0607	0.8318	0.2224	23.5486	0.8542	0.1444
	50K	23.2573	0.8359	0.2114	23.8038	0.8572	0.1388

**Table 4:** Comparison of the algorithms based on the rendering quality metrics in study area 2.

Method	Number of Iterations	Downscale (4)			Downscale (8)		
		**PSNR**	**SSIM**	**LPIPS**	**PSNR**	**SSIM**	**LPIPS**
	5K	19.9150	0.4104	0.6216	20.8616	0.5137	0.3584
Nerfacto	30K	20.0619	0.4233	0.4746	20.6049	0.5018	0.2466
	50K	18.7324	0.3550	0.4482	20.5677	0.4954	0.2296
	5K	21.6822	0.4952	0.5761	23.2117	0.6546	0.3547
Instant-NGP	30K	22.7156	0.5703	0.4610	24.6328	0.7586	0.2535
	50K	22.9457	0.5857	0.4397	24.7991	0.7715	0.2399
	5K	23.3103	0.6733	0.4077	24.4501	0.7713	0.2512
Splatfacto	30K	27.6840	0.8898	0.0959	28.9073	0.9186	0.0589
	50K	27.5989	0.8887	0.0919	28.9315	0.9180	0.0561

## Data Availability

Data set available on request from the author.

## Abbreviations

The following abbreviations are used in this manuscript:

UAV	Unmanned Aerial Vehicle
NeRF	Neural Radiance Field
3DGS	Three-dimensional gaussian splatting
3D	Three-dimensional
AI	Artificial intelligence
SfM	Structure-from-Motion
MVS	MultiView Stereo
M3C2	Multiscale Model-to-Model Cloud Comparison
LiDAR	Light Detection and Ranging
TDOM	True digital orthophoto map
RTK	Real time kinematic
5D	Five-dimensional
GPU	Graphics processing unit
VR	Virtual reality
SIFT	Scale-invariant feature transform
PnP	Perspective-n-points
PSNR	Peak signal-to-noise ratio
SSIM	Structural similarity index measure
LPIPS	Learned perceptual image patch similarity
MSE	Mean square error
ICP	Iterative closest point
GCP	Ground control point
